# Methanolic Root Extract of *Rauwolfia serpentina* Benth Improves the Glycemic, Antiatherogenic, and Cardioprotective Indices in Alloxan-Induced Diabetic Mice

**DOI:** 10.1155/2012/376429

**Published:** 2012-12-18

**Authors:** Muhammad Bilal Azmi, Shamim A. Qureshi

**Affiliations:** ^1^Department of Biochemistry, University of Karachi, Karachi 75270, Pakistan; ^2^Quality Enhancement Cell, Dow University of Health Sciences, Karachi 74200, Pakistan

## Abstract

The aim of the study was to evaluate the phytochemistry and the effect of methanolic root extract (MREt) of *Rauwolfia serpentina* on alloxan-induced diabetic *Wister* male mice. Mice were divided in control (distilled water at 1 mL/kg) and alloxan-induced diabetic mice which subdivided into diabetic (distilled water at 1 mL/kg), negative (0.05% dimethyl sulfoxide at 1 mL/kg), positive (glibenclamide at 5 mg/kg) controls, and three test groups (MREt at 10, 30, and 60 mg/kg). All treatments were given orally for 14 days. Qualitatively MREt showed the presence of alkaloids, carbohydrates, flavonoids, glycosides, cardiac glycosides, phlobatannins, resins, saponins, steroids, tannins, and triterpenoids, while quantitatively extract was rich in total phenols. The flavonoids, saponins and alkaloids were also determined in root powder. MREt found effective in improving the body weights, glucose and insulin levels, insulin/glucose ratio, glycosylated and total hemoglobin in test groups as compared to diabetic control. Similarly, significantly decreased levels of total cholesterol, triglycerides, low-density lipoprotein (LDL-c), and very low-density lipoprotein (VLDL-c) cholesterols were found in test groups. Significant lipolysis with improved glycogenesis was also found in liver tissues of all test groups. ALT levels were found normal in all groups. Thus, MREt improves the glycemic, antiatherogenic, coronary risk, and cardioprotective indices in alloxan-induced diabetic mice.

## 1. Introduction

Today more than 385 million people are suffering from diabetes worldwide and forecasted that 439 million adults will develop this disease in 2030 with high prevalence in developing countries [1]. Similarly, Pakistan is facing the same problem, and it will be ranked fourth among countries with 14.5 million people having diabetes in 2025 [2]. Therefore, diabetes due to absolute or relative insulin deficiency or insulin resistance becomes a widespread endocrine disorder that not only affects the glucose homeostasis but also chronically alters lipid and protein metabolisms with increase in cellular oxidative stress [3]. 

Commercially available pharmaceutical formulations used for the treatment of diabetes are not entirely free from side effects and do not completely restore normal glucose homeostasis [3]. On the contrary, plant-based medicines are water soluble with no side effect. It has been reported that over 80%, world population is dependent on herbal medicine for their therapeutic benefits [4], and more than 800 plant species have been mentioned in the literature with significant hypoglycemic activity [5]. However, searching for new antidiabetic drug from natural sources including herbs is still an attractive research aspect as these are cost effective substances with no side effect. Most of herbal medicines contain glycosides, alkaloids, terpenoids, flavonoids, carotenoids, and so forth that have significant hypoglycemic effect [6, 7]. Therefore, plant kingdom has become a target for multinational drug companies and research institutes for the discovery of new biologically active compounds that could be potential antidiabetic drug with few or no side effects. 

Medicinally important herb *Rauwolfia serpentina *Benth (family: Apocynaceae) has an extensive spectrum of valuable therapeutic actions with mainly effective in the treatment of hypertension and psychotic disorders like schizophrenia, anxiety, insomnia, insanity, and so forth [8, 9]. It has also reported in the treatment of skin cancers, burns, eczema, and snake bite [10, 11]. Various indole alkaloids and related constituents have been isolated from the roots of this plant which have significant biological activities [12]. The root extract was found effective in the treatment of gastrointestinal disorders like diarrhea, dysentery, cholera, and so forth and also in breast cancer [11, 13]. An *in vitro* study described the antimicrobial and antioxidant activities of leaf extract of this plant [11]. The hypotensive action of *R. serpentina, *in terms of reserpine one of its major constituents that is commonly used as a natural tranquilizer, has been well scientifically investigated and documented [14] as compared to other briefly described activities such as hypoglycemic activity. Though, therapeutic effects of *Rauwolfia* with incomplete hypoglycemic action in diabetic patients, diabetic hypertensive patients and in anesthetized cats were brief [15–17]. A preliminary study related to its hypoglycaemic and hypolipidemic activities in alloxan-induced diabetic rats has been published in 2009 by using a single dose of methanolic root extract of *R. serpentina *[18] which was further elaborated by determining the acute toxicity and median lethal dose (LD_50_) of same extract [19]. Therefore, in continuation of this research idea, the present study was designed to evaluate the phytochemistry and long-term therapeutic effect of methanolic root extract of *R. serpentina *on glycemic, antiatherogenic, and cardioprotective indices in alloxan-induced diabetic mice.

## 2. Materials and Methods

### 2.1. Plant Material

Roots of *Rauwolfia serpentina *were purchased from Hamdard Dawakhana, Saddar, Karachi and identified by expert in Botany Department, University of Karachi, Karachi-75270, Pakistan. The voucher specimen has been kept in our department (KU/BCH/SAQ/02).

### 2.2. Preparation of Methanolic Root Extract (MREt)

Forty grams of ground powder of roots of *R. serpentina *was extracted with methanol (1 L; 95%) overnight and filtered through Whatman no. 1 filter paper twice. The filtrate was then concentrated till dryness by using rotary vacuum evaporator (Eylea-18) to obtain brown residue that referred as methanolic root extract [18].

### 2.3. Phytochemical Analyses 

#### 2.3.1. Qualitative Phytochemical Analysis of MREt

The MREt of *R. serpentina* was tested for the presence of different phytoconstituents like alkaloids, flavonoids, and so forth by standard methods [20–22].

**(1) Tests for Alkaloids**

- *Hager's Test.* MREt (1 mL) was taken in the test tube and few drops of Hager's reagent were added, resulted in the formation of yellow precipitate which confirmed the presence of alkaloids.
- *Wagner's Test.* MREt (1 mL) was acidified with hydrocholoric acid, to this few drops of Wagner's reagent was added. A yellow or brown precipitate indicated the presence of alkaloids.

**(2) Test for Anthraquinones (Borutrager's Test)**

To MREt (1 mL), 10% FeCl_3_ (1 mL) and concentrated HCl (0.5 mL) were added. Boiled in a water bath for few minutes, filtered it and the filtrate was treated with diethyl ether and concentrated ammonia. Appearnce of pink or deep colour indicated the presence of anthraquinones.

**(3) Tests for Carbohydrates**

- *Benedict's Test.* MREt (2 mg) was shaken with distilled water (10 mL), filtered and filtrate was concentrated, then Benedict's reagent (5 mL) was added to this and boiled for 5 minutes, resulted in the formation of the brick red color precipitate within 5 minutes and indicated the presence of carbohydrates.
- *Fehling's Test.* MREt (2 mg) was shaken with distilled water (10 mL), filtered and filtrate was concentrated, to this mixture of the equal parts of the Fehling's solution A and B (1 mL) were added and boiled for few minutes, resulted in the formation of red or brick color precipitate which indicated the presence of the reducing sugar.
- *Mohlisch's Test.* MREt (2 mg) was shaken with distilled water (10 mL), filtered and filtrate was concentrated, to this 2 drops of freshly prepared alcoholic solution of *α*- naphthol (20%) and concentrated sulphuric acid (2 mL) were added to observe a layer below the mixture. Red violet ring appeared that indicated the presence of carbohydrates which disappeared on the addition of the excess alkali. The same test was also used to detect the presence of glycosides in extract.

**(4) Test for Flavonoids (Ammonia Test)**

A small piece of filter paper was dipped in MREt (1 mL) and exposed to ammonia vapours that resulted in the formation of yellow spot on filter paper indicated the presence of flavonoids.

**(5) Test for Cardiac Glycosides (Keller-Killani Test)**

MREt (5 mL) was treated with glacial acetic acid (2 mL) mixed with one drop of ferric chloride solution. To this concentrated sulphuric acid (1 mL) was added. A brown ring appeared at the interface indicated a deoxy sugar characteristic of cardenolides, followed by the formation of a violet ring below that brown ring.

**(6) Test for Resins**

MREt (1 mL) was dissolved in acetone (1 mL), and the solution was poured in distilled water (2 mL). Turbidity indicated the presence of resins.

**(7) Test for Steroids (Liebermann-Burchard's Test)**

MREt (0.5 mL) was dissolved in acetic anhydride, heated to boiling, cooled, and then concentrated sulphuric acid (1 mL) was added along the sides of test tube that resulted in the formation of green color indicated the presence of the steroids.

**(8) Test for Saponins**

MREt (5 mL) was taken in test tube and a drop of the sodium bicarbonate solution was added, shaked vigorously, and left for 3 minutes. Formation of the honeycomb like froth indicated the presence of saponins.

**(9) Test for Triterpenoids (Salkowski's Test)**

MREt (5 mL) was shaken with chloroform (2 mL) followed by the addition of sulphuric acid (3 mL) slowly by the sides of the test tube. Formation of the reddish brown color indicated the presence of the steroids.

**(10) Test for Tannins**

To MREt (1–2 mL), few drops of FeCl_3_ (5%) solution was added resulted in the formation of green colour indicated the presence gallotannins, while brown color indicated the presence of pseudotannins.

**(11) Test for Phlobatannins**

MREt (1 mL) was boiled with hydrochloric acid (1%) resulted in the formation of red precipitate which indicated the presence of phlobatannins.

#### 2.3.2. Quantitative Phytochemical Analysis

**(1) Determination of Alkaloids**

Ground root powder (5 g) of *R. serpentina* was weighed and taken into a beaker (250 mL). To this 10% acetic acid in ethanol (200 mL) was added, covered, and allowed to stand for 4 hours. This was filtered and the extract was concentrated on a water bath to 1/4 of its original volume. Concentrated ammonium hydroxide was added drop wise to the extract until the precipitation was completed. The whole solution was allowed to settle and the precipitate was collected and washed with dilute ammonium hydroxide and then filtered. The residue is the alkaloid, which was oven-dried and weighed [23].

**(2) Determination of Flavonoid**

Ground root powder (10 g) of *R. serpentina* was extracted repeatedly with 80% aqueous methanol (100 mL) at room temperature. The whole solution was filtered through Whatman filter paper no. 42 (125 mm). The filtrate was later transferred into a crucible and evaporated into dryness over a water bath and weighed untill a constant weight was achieved [24].

**(3) Determination of Saponin**

Ground root powder (20 g) of *R. serpentina* was taken into a conical flask and 20% aqueous ethanol was added. The sample was heated over a hot water bath for 4 hours with continuous stirring at about 55°C. The mixture was filtered and the residue reextracted with another 200 mL of same 20% ethanol. The combined extracts were reduced to 40 mL over water bath at about 90°C. The concentrate was transferred into a 250 mL separatory funnel and 20 mL of diethyl ether was added and shaken vigorously. The aqueous layer was recovered while the diethyl ether layer was discarded. The purification process was repeated. 60 mL of n-butanol was added. The combined n-butanol extracts were washed twice with 5% aqueous sodium chloride (10 mL). The remaining solution was heated in a water bath. After evaporation, the sample was dried in the oven to a constant weight [25].

**(4) Determination of Total Phenolic Content**

Total phenolic content was determined using Folin-Ciocalteu reagent. Test, having 0.1 mg of the plant extract, was prepared and mixed with 1 mL of Folin-Ciocalteu reagent (1 : 10 diluted with distilled water) after 3 minutes, 2% sodium carbonate (3 mL) was added, shaken for few seconds, and then incubated for 2 hours at room temperature with intermittent shaking. The absorbance was read at 760 nm against a reagent blank. The standard curve was prepared using 0.01–0.1 mg of gallic acid. The total phenolic content was expressed in mg/g of MREt [26].

### 2.4. Alloxan Monohydrate (Sigma)

Diabetes was induced in overnight fasted mice by a single intraperitoneal injection of alloxan monohydrate (150 mg/kg). After 72 hours of this injection fasting blood glucose levels were monitored from tail vein of mice with the help of glucometer (*Optium Xceed*, Diabetes Monitoring system by Abbott), mice showed glucose level ≥190 mg/dL were selected, grouped, and each group subjected to its respective treatment.

### 2.5. Glibenclamide

Antidiabetic drug glibenclamide (*Doanil*) purchased from Sanofi-Aventis Pakistan Ltd. and used as positive control (5 mg/kg). 

### 2.6. Dimethyl Sulphoxide (DMSO)

An analytical grade DMSO was purchased from Fisher Scientific (UK), and its 0.05% concentration was used as vehicle for administering the doses of MREt in test mice. 

### 2.7. Experimental Mice and Treatment Groups

Male albino *Wister* mice (25–35 grams) were purchased from the breeding house of Dow University of Health Sciences (DUHS), Karachi. Experimental mice were acclimatized and maintained individually in cages in an air conditioned room with temperature not more than 23 ± 2°C (relative humidity 55%) for one week prior to the experiment in the conventional animal house of same university. The care and handling of these mice were in accordance to the internationally accepted standard guidelines for animal handling. Mice were given standard laboratory diet with free access to water *ad libitum *and during the experimental period no physical stress was provided. The experimental protocol was approved by the Institutional Ethical Review Board (IERB) of DUHS in June 2010 (Authority letter Reference Number: IRB-186/DUHS-10).

Overnight (12–14 hours) fasted mice (blood glucose level ≥190 mg/dL) were randomly divided into seven groups (6 mice/group) in the following manner.

1. Group I: control: normal mice treated with distilled water (1 mL/kg).
2. Group II: diabetic control: alloxan-induced diabetic mice treated with distilled water (1 mL/kg).
3. Group III: negative control: alloxan-induced diabetic mice treated with 0.05% DMSO (1 mL/kg).
4. Group IV: positive control: alloxan-induced diabetic mice treated with glibenclamide (5 mg/kg).
5. Group V: test group: alloxan-induced diabetic mice treated with MREt (10 mg/kg).
6. Group VI: test group: alloxan-induced diabetic mice treated with MREt (30 mg/kg).
7. Group VII: test group: alloxan-induced diabetic mice treated with MREt (60 mg/kg).

Each treatment was given to its respective group orally once in a day for 14 days consecutively. At the end of animal trial, mice were sacrificed to collect whole blood, serum and liver tissues that were used to analyze hematological and biochemical parameters. 

### 2.8. Determination of Body Weight

Body weights of animals of each group were measured at initial (0 day) and final (14 day) days of trial by using balance (Kitchen scale 1800) and calculate percentage of body weight gain or loss with the following formula [27]:

(1) %  Body  weight  change  =Final  weight−initial  weightinitial  weight×100.

### 2.9. Determination of Biochemical Parameters

Fasting blood glucose levels were monitored in each group by glucometer at initial (0 day) and final day (14 day) of trial to determine percent glycemic change by using the following formula [28]. Where *G*_*o*_ = blood glucose level at 0 day and *G*_*x*_ = blood glucose level at 14 day:

(2) $$\begin{array}{c} \%\;\text{Glycemic}\;\text{change}=\frac{G_{x}-G_{o}}{G_{o}}\times 100. \end{array}$$

Other parameters including serum alanine aminotransferase (ALT), total cholesterol (TC), triglycerides (TG), high density lipoprotein-cholesterol (HDL-c) concentrations were determined by commercially available enzymatic assay kits (*Randox*, United Kingdom). Whereas low density lipoprotein-cholesterol (LDL-c) and very low density lipoprotein-cholesterol (VLDL-c) were calculated by Friedewald formulae [29]. Estimation of total lipids and glycogen contents in liver homogenate was done by gravemetric and colorimetric methods,respectively [30, 31].

### 2.10. Determination of Serum Insulin Level and Insulin/Glucose (I/G) Ratio

The serum insulin levels were determined by cobas e411 analyzer, Hitachi (Roche Diagnostics GmbH, Mannheim, Germany) and expressed as *μ*U/mL. Whereas I/G ratio was determined as fasting insulin (*μ*U/mL)/glucose (mg/dL) and expressed as *μ*U/mg [32].

### 2.11. Determination of Hematological Parameters

Total hemoglobin (Hb) and glycosylated hemoglobin level (HbA1_c_) levels were evaluated by Automated Analyzer, Sysmex (XS-1000i) and commercially available Kit (Nycocard Kit, USA), respectively. 

### 2.12. Determination of Antiatherogenic, Cardioprotective, and Coronary Risk Indices

Cardioprotective index (CPI) was estimated in term of HDL-c to LDL-c and TG to HDL-c ratios [33, 34].Where as antiatherogenic (AAI) and coronary risk indices (CRI) were calculated by the following formulae [35, 36]:

(3) $$\begin{array}{c} \text{AAI}=100\times \left[\frac{\text{HDL-c}}{\text{Total}\;\text{cholesterol}-\text{HDL-c}}\right],\\ \text{CRI}=\frac{\text{Total}\;\text{cholesterol}}{\text{HDL-cholesterol}}. \end{array}$$

### 2.13. Statistical Analysis

Results of the present study are expressed as mean ± SEM (standard error mean). The data were analyzed with statistical package for social sciences (SPSS version 18) by using *one-way *ANOVA followed by LSD (least significant difference) test at *P* < 0.05. The differences were considered significant at *P* < 0.05, *P* < 0.01, *P* < 0.001, and *P* < 0.0001 when compared with respective controls.

## 3. Results

### 3.1. Total Yield of MREt of **R. serpentina**

Total yield of MREt of *R. serpentina* was 5% g/g of dry root powder. The quality of extract was maintained by kept in an air-tight container and stored in refrigerator below 10°C until used.

### 3.2. Phytochemical Profile of MREt

Qualitative analysis of MREt showed the presence of alkaloids, carbohydrates, flavonoids, glycosides, cardiac glycosides, phlobatannins, resins, saponins, steroids, tannins, and triterpenoids. Whereas quantitatively the amount of alkaloids, saponins, flavonoids in root powder and total phenols in MREt were found as 7, 97, 20 and 233 mg/gm respectively (Table 1). 

### 3.3. Effect of MREt on Body Weight

In diabetic and negative controls marked reduction in percent body weight was observed up to −11.08 and −9.25%, respectively as compared to control mice. Whereas +8.45% gain in body weight was observed in mice treated with glibenclamide as compared to diabetic and negative controls (*P* < 0.0001). Though all three doses of MREt slightly reduced (*P* < 0.05 and *P* < 0.01) the body weight in test mice but it was not as high as it was observed in diabetic and negative controls (Table 2). 

### 3.4. Effect of MREt on Blood Glucose Level

Significant percent glycemic change was found as −51, −46, and −49% in test mice treated with MREt at 10, 30, and 60 mg/kg, respectively as compared to diabetic and negative controls (*P* < 0.0001). Similarly −40% reduction in blood glucose level was also observed in positive control group (Table 3).

### 3.5. Effect of MREt on Hb and *HbA*1_*c*_

**Figure 1:**
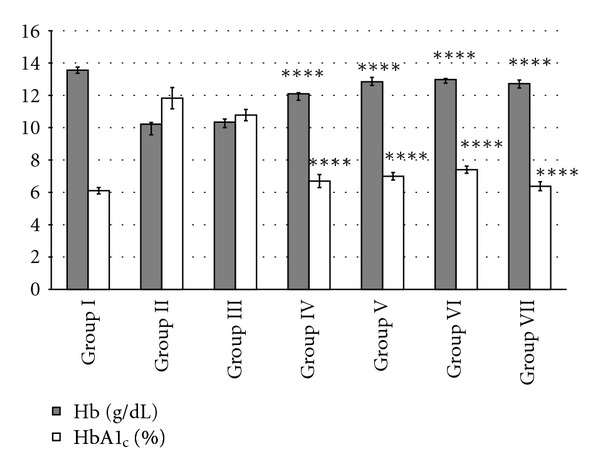
Effect of MREt on hemoglobin (g/dL) and glycosylated hemoglobin (HbA1_c_) levels. Each bar represents the mean ± SEM (*n* = 6).   ****P < 0.0001, when compared with group II and III.

Glibenclamide andall three doses of MREt significantly (*P* < 0.0001) improved the total Hb level from 12.10 to 12.98 g/dL in positive control and test groups as compared to diabetic and negative control mice that showed 10 g/dL (Figure 1). Similarly significantly improved percentage of   HbA1_c_ from 6.3 to 7.4% was observed in all test groups (*P* < 0.0001) and positive control as compared to same controls that showed its value from 10.78 to 11.83% (Figure 1). 

### 3.6. Effect of MREt on Lipid Profile

MREt at 10, 30, and 60 mg/kg in test mice induced significant (*P* < 0.0001) decrease in serum TC levels up to 146.19, 129.33, and 138.50 mg/dL, respectively as compared to diabetic and negative controls which showed TC levels up to 260.42 and 254.11 mg/dL, respectively. Glibenclamide treated group also showed significant (*P* < 0.0001 & *P* < 0.001) decrease up to 178.40 mg/dL as compared to same controls (Table 4). A significant decrease was also found in serum TG levels up to 128.27 and 138.75 mg/dL in test mice treated with same extract at 10 and 60 mg/kg, respectively as compared to diabetic and negative control groups that showed the same parameter up to 161.33 and 173.50 mg/dL (*P* < 0.05 and *P* < 0.01). Though MREt at 30 mg/kg also decreased the TG level in its respective group as compared to same control groups but it was not statistically significant. Prominent fall (*P* < 0.05 and *P* < 0.01) was also observed in same parameter of positive control group (Table 4). LDL-c levels in diabetic test mice treated with MREt at 10, 30 and 60 mg/kg were significantly decreased (*P* < 0.01, *P* < 0.001 and *P* < 0.0001) up to 83.83, 63.46, and 73.30 mg/dL, respectively as compared to diabetic and negative controls that showed very high levels of same parameter (Table 4). Decrease (*P* < 0.05 and *P* < 0.01) levels of VLDL ranging from 24 to 29 mg/dL was also observed in positive control and three test groups (Table 4). However, decreased levels of HDL-c were observed in test mice as compared to control, diabetic, and negative control groups but that decrease was in relation of low levels of TC that were observed in test mice (Table 4).

### 3.7. Effect of MREt on CPI, AAI, and CRI

**Figure 2:**
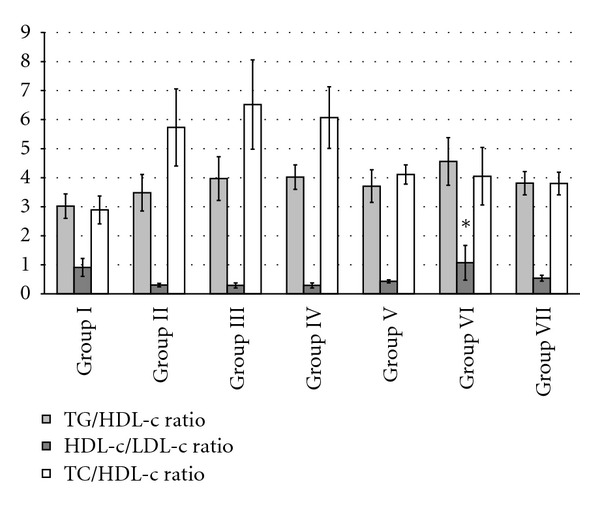
Effect of MREt on cardioprotective indices. Each bar represents the mean ± SEM (*n* = 6).   *P < 0.05, when compared with group II and III.

In case of CPI, TG/HDL-c ratio was found reduced from 3.71 to 4.56 in positive control and test groups treated with glibenclamide and MREt, respectively (Figure 2). Similarly improved HDL-c/LDL-c ratio was observed in test groups from 0.43 to 1.07 compared with diabetic and negative controls which showed reduced same ratio from 0.29 to 0.3 (Figure 2). CRI in term of TC/HDL-c ratio was observed decrease in test groups treated with MREt from 3.8 to 4.11 as compared to same control groups which showed marked increase from 5.73 to 6.52 in the same ratio. Even positive control group showed high value of same index (Figure 2). An improvement was also found in values of AAI from 33 to 53% in test groups as compared to diabetic, negative, and positive controls that showed the same AAI from 23 to 24% (Table 4). 

### 3.8. Effect of MREt on ALT

Normal serum ALT levels were observed in all experimental controls and test groups (Table 4). 

### 3.9. Effect of MREt on Liver Glycogen and Total Lipids

**Figure 3:**
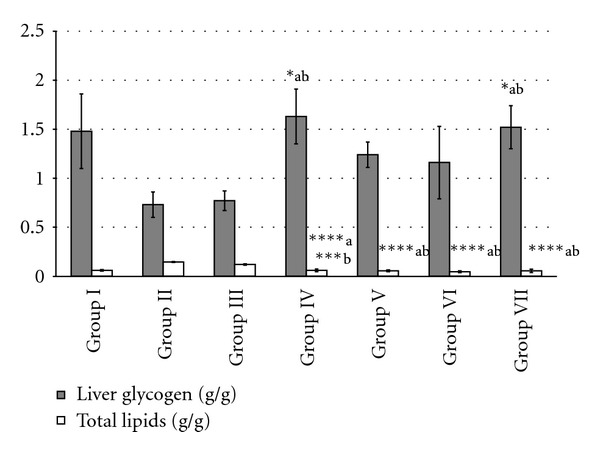
Effect of MREt on hepatic glycogen and total lipids (g/g). Each bar represents the mean ± SEM (*n* = 6).   *P < 0.05 and  ****P < 0.0001, when compared with group II (a) and (b) III.

The liver glycogen amount was found improved in positive control and test groups from 1.16 to 1.63 g/g of tissue as compared to diabetic and negative control groups that showed its decrease amount from 0.73 to 0.77 g/g tissue (Table 4). Whereas total lipid content in liver tissue was increased in diabetic and negative control groups (0.145 ± 0.005 and 0.12 ± 0.007 g/g tissue) as compared to MREt treated mice and positive control group that showed significantly reduced (*P* < 0.0001) level of total lipids in their livers from 0.045–0.06 g/g of tissue (Figure 3). 

### 3.10. Effect of MREt on Serum Insulin Level and Insulin/Glucose (I/G) Ratio

**Figure 4:**
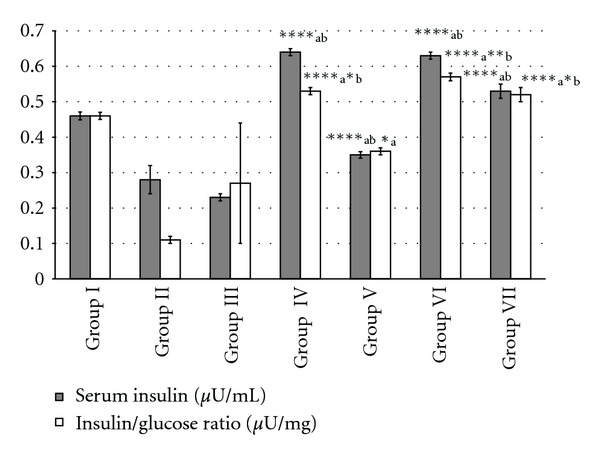
Effect of MREt on serum insulin level and insulin/glucose ratio. Each bar represents the mean ± SEM (*n* = 6).   *P < 0.05,  **P < 0.01, and  ****P < 0.0001, when compared with group II (a) and III (b).

Serum insulin levels and I/G ratio were significantly improved (*P* < 0.05, *P* < 0.001 and *P* < 0.0001) in all test and positive control groups and found 0.35–0.64 *μ*U/mL and 0.36–0.57 *μ*U/mg, respectively as compared to diabetic and negative controls that showed low levels of serum insulin and I/G ratios (Figure 4).

## 4. Discussion

The frequency of diabetes increases day by day with rapid rise in mortality burden that establishes threat of this disease as one of the leading cause of death globally [1]. Beside the presence of commercially available oral antidiabetic drugs and insulin injections for the treatments of both type I and II diabetes, a vast scientific data is available which describe that variety of herbs are used as effective hypoglycemic agents with different modes of action [37]. Today investigating a hypoglycemic herb with potent hypotensive and hypolipidemic activities is a strong and interesting research aspect of ethanopharmacology. The detailed spectrum of *Rauwolfia serpentina* in the management of diabetic dyslipidemia has not been reported yet. Therefore the present study was designed to identify the phytochemical profile of MREt of *R. serpentina* and its long-term hypoglycemic, hypolipidemic effects and weight improving pattern in alloxan-induced daibetic *Wister* male mice. 

 The present study demonstrates the presence of alkaloids, carbohydrates, flavonoids, glycosides, cardiac glycosides, phlobatannins, resins, saponins, steroids, tannins, and triterpenoids in MREt. The quantitative profile of the same extract clearly illustrates the presence of high magnitude of antioxidant compounds like total phenols 233 mg/gm of extract. Similarly, flavonoids, alkaloids, and saponins were found as 97, 7, and 20 mg/gm in root powder of same herb. A vast literature provides scientific evidence that plants rich in flavonoids possess potent antidiabetic, hypolipidemic, hypotensive, anti-inflammatory, and antioxidative activities [5, 38]. 

Alloxan is a pyrimidine derivative and commonly used as universal toxin that causes the irreversible destruction of *β*-cells in the islets of Langerhans, which not only induced diabetes by inhibiting the release of insulin but also act as a good enhancer of oxidative stress by elevating the production of reactive oxygen species (ROS) that directly associated with the weight loss in diabetic mice [39]. However, in the present investigation, the percent change in body weights of alloxan-induced diabetic mice treated with the doses of MREt of *R. serpentina* showed less reduction than diabetic control groups at 14 day of animal trial. Of which, MREt in a dose of 60 mg/kg showed marked improvement in body weight whereas the body weights recovered slowly in the other two test groups treated with 10 and 30 mg/kg of same extract. In diabetic condition, body utilized triglycerides as an alternate source of energy which is also accompanied by catabolism of tissue protein that results in loss of both fat and lean mass which in turn induce a significant reduction in total body weight [40]. It has been reported that this unhealthy loss in total body weight is more accelerated due to polyuria induced by dehydration because of hyperglycemia, one of the characteristic symptoms of diabetes [41]. The MREt (10, 30, and 60 mg/kg) induced a significant percent glycemic reduction in fasting blood glucose levels of all three test groups at 14 day from −46 to −51% as compared to marked increase in percent glycemic change from 23 to 28% that was observed in diabetic and negative control groups. This observation also supports our previous finding that MREt of *R. serpentina* improves the glucose tolerance in *Wister* mice [19].

The antidiabetic effect of MREt of *R. serpentina* is more strengthened by observing the fair control of HbA1_c_ levels in all three test groups treated with the same extract and interestingly, the HbA1_c_ levels in test groups at 14 day of trial were found almost equal to its level observed in normal (control) and positive control mice. However, the high percentage of HbA1_c_ was found in diabetic and negative controls that represents poor glycemic control. It has been reported that in diabetes, increased amount of blood glucose nonezymatically combines with hemoglobin to form glycated (glycosylated) hemoglobin (HbA1_c_), and hence the HbA1_c_ level reflects the average amount of glucose in blood [42]. Increased concentration of HbA1_c_ in diabetes also affects the total Hb level in blood besides the contribution of other chronic causes such as renal dysfunction, and in this regard several studies reported the presence of unrecognized anemia in diabetic patients [43]. In the present study, MREt significantly improved the total Hb levels in all test groups and brought it back to the level that was observed in control mice, as compared to diabetic and negative controls that showed low levels of same parameter. 

The elevated serum levels of TC, TG, and LDL-c provide a high risk for the development of atherosclerosis and other cardiovascular diseases (CVD) whereas increased HDL-c level associated with the decrease in this risk [40]. In alloxan-induced diabetes, hyperglycemia is associated with hypertriglyceridemia and hypercholesterolemia [18]. In the present study, MREt of *R. serpentina *significantly decreases the levels of TC, TG, LDL-c, and VLDL-c in all test groups as compared to the diabetic and negative control groups. Both lipoproteins including LDL-c and VLDL are involved in depositing TC and TG on walls of coronary arteries and initiate the process of atherosclerotic plaques [44]. Reduced serum levels of LDL-c and VLDL-c found in test mice groups treated with doses (10, 30, and 60 mg/kg) of MREt, is among one of the beneficial aspects of this current research and proved the antiatherosclerotic potential of this extract. However, a decrease in HDL-c levels was also observed in test mice, which actually reflects the significantly lower levels of TC found in test groups as only 30% cholesterol transported by HDL-c in blood from peripheral tissues to liver for its metabolism and excretion [45]. Scientific evidence described that many plant extract or herbal products possess lipid lowering potential by producing the decrease in all cholesterol-associated lipoprotein levels including HDL-c but improving the cardioprotective indices [46]. Hypocholesterolemic and hypotriglyceridemic effects of MREt are actually the result of its potent hypoglycemic effect that is probably due to an increase in glucose utilization by peripheral tissues [18] or inhibiting the activity of rate-limiting enzyme 3-hydroxy-3-methyl glutaryl CoA reductase (HMG-CoA reductase) of cholesterol biosynthesis. The experimentally obtained hypotriglceridemic effect of MREt may also be due to the improvement in lipolysis by reducing the activity of hormone-sensitive lipase [40, 47]. 

To support lipid lowering potential of MREt of *Rauwolfia*, the antiatherogenic index (AAI) was also evaluated and found increased from 33 to 53% in test groups mice treated with extract (10, 30, and 60 mg/kg) as compared to diabetic controls which again represents the antiatherosclerotic significance of *Rauwolfia*. Slight improvement has also been observed in cardioprotective index of test groups in terms of TG/HDL-c and HDL-c/LDL-c ratios as compared to diabetic control. TG/HDL-c ratio is preferable under 4 and ideal under 2 whereas HDL-c/LDL-c ratio is preferable over 0.3 and ideal over 0.4 [48]. Out of these ratios, HDL-c/LDL-c ratio was found ideal in the present study. It has been reported that an increase in TG/HDL-c and a decrease in HDL-c/LDL-c ratios are good predictors of CVD and insulin resistance in subjects [49]. Similarly coronary risk index (CRI) in term of TC/HDL-c ratio significantly decreased in all MREt treated test mice, which provides further strength to the present study. According to the past findings, the value of CRI greater than 5 indicates that subject is on high risk of CVD while under 3.5 is ideal for biochemical functioning [33, 34]; hence, CRI is highly improved in MREt treated test mice. 

Attention-grabbing point of the present study is that the oral antidiabetic drug glibenclamide, one of the well-known sulfonylureas, also produced significant antidiabetic effect in alloxan-induced diabetic mice though this drug is classified as secretagogues and it requires alive *β*-cells of pancreas to enhance the release of insulin [3]. It was also proved by observing the increase in serum insulin level and insulin/glucose (I/G) ratio in positive control group which constitutes alloxan-induced diabetic mice treated with glibenclamide at 5 mg/kg, it tells that may be few *β*-cells were still alive. Though, extrapancreatic effects of sulfonylureas in type-II diabetes have also been reported [50]. Similarly, the serum insulin level and I/G ratio were gradually improved in all MREt treated mice (test groups) and become better than its levels found in diabetic control group that showed decreased insulin level and increased I/G ratio. Interestingly this improvement provides another possibility of antidiabetic mechanism of action of MREt that besides having extrapancreatic action, it may also has some pancreatic action as same as glibenclamide through which it could enhance the release of insulin via activating few alive *β*-cells as a result of which insulin in turn produce its anabolic action by improving the ability of body to utilize glucose as a source of energy. This possibility was also supported by observing the gradual improvement in liver glycogen in all test mice treated with MREt from 10 to 60 mg/kg and even the highest experimental dose of extract gave same amount of hepatic glycogen that was observed in control group whereas its low amount was observed in diabetic and negative control groups. This finding clearly proved that the process of glycogenesis has been improved in all test mice by *Rauwolfia*. Similarly the total lipids in liver tissue were significantly recovered in MREt treated test groups as compared to diabetic and negative controls which indicate the hepatoprotective effect of this medicinal plant as reduction in liver lipid content improves the liver functioning [51]. 

ALT is liver-specific enzyme that provides a deep insight about the hepatic functioning, it elevates in case of hepatic injuries or malfunctioning [44]. The pharmacological basis of therapeutics clearly states that orally administrated drug must pass through hepatic metabolism so it should not be toxic to liver [18].Therefore, to investigate any toxic effect of MREt on liver, serum ALT level was measured. Though, the decreased levels of ALT were observed in all test groups treated with MREt (10, 30, and 60 mg/kg) as compared to normal, diabetic and negative controls but considered normal levels of this enzyme. As scientific literature states that normal ALT level range from 0 to 37 U/L [52, 53]. 

The obtained significant antidiabetic and hypolipidemic effects of MREt of *R. serpentina* may be due to the presences of high quantity of total polyphenolic compounds in the same extract as compared to other constituents. Therefore, total flavonoids could be targeted, isolated, and studied as an active fraction involved in antidiabetic activity of same extract in future. 

## 5. Conclusion

The present study concludes that MREt of *R. serpentina* is an effective antidiabetic agent as it improves glycemic, antiatherogenic, and cardioprotective indices in alloxan-induced diabetic mice. 

## Figures and Tables

**Table 1:** Phytochemical analysis of MREt of *R. serpentina*.

Phytoconstituent	Qualitative analysis	Quantitative analysis
		(mg/gm)
Alkaloids	Positive	7*
Anthraquinones	Negative	—
Carbohydrates	Positive	—
Flavonoids	Positive	97*
Glycosides	Positive	—
Cardiac glycosides	Positive	—
Phlobatannins	Positive	—
Resins	Positive	—
Saponins	Positive	20*
Steroids	Positive	—
Tanins	Positive	—
Triterpenoids	Positive	—
Total phenols	Positive	233

*mg/gm of root powder.

**Table 2:** Effect of MREt of *R. serpentina* on body weights of mice.

Groups	Treatments	Body weight (gm)		Weight change (%)
		Initial weight at 0 day	Final weight at 14 day
Group I	Distilled water (1 mL/ kg)	28.25 ± 0.85	31.25 ± 0.85	+10.68 ± 1.49
Group II	Alloxan treated (150 mg/kg)	29.25 ± 1.6	26 ± 1.41	−11.08 ± 1.48
Group III	0.05% DMSO (1 mL/kg)	29.5 ± 0.87	26.75 ± 0.63	−9.25 ± 0.62
Group IV	Glibenclamide (5 mg/kg)	29.5 ± 0.5	32 ± 0.71	+8.45 ± 0.89^∗∗∗∗a,b^
Group V	MREt (10 mg/kg)	30.75 ± 1.31	29 ± 1.22	−5.69 ± 0.78^∗a^
Group VI	MREt (30 mg/kg)	28.50 ± 1.55	26.75 ± 1.65	−6.25 ± 1.05^∗a^
Group VII	MREt (60 mg/kg)	27.50 ± 1.5	26.75 ± 1.89	−2.94 ± 2.37^∗∗∗∗a,∗∗b^

Values are expressed as mean ± SEM (*n* = 6). *P < 0.05, **P < 0.01, and ****P < 0.0001, when compared with respective group II (a) and III (b). Negative (−)/positive (+) signs represent loss/gain in weights of mice at 14th day.

**Table 3:** Effect of MREt of *R. serpentina* on fasting blood glucose level in mice.

Groups	Treatments	Initial day (*G*_0_)	Final day (*G*_*x*_)	Glycemic change (%)
Group I	Distilled water (1 mL/ kg)	102.25 ± 2.72	98 ± 5.84	−4.16
Group II	Alloxan treated (150 mg/kg)	198.25 ± 4.91	245.50 ± 20.66	23.83
Group III	0.05% DMSO (1 mL/kg)	199.50 ± 5.62	257.25 ± 22.68	28.95
Group IV	Glibenclamide (5 mg/kg)	195.25 ± 3.12	116.50 ± 14.46****	−40.46
Group V	MREt (10 mg/kg)	198.25 ± 5.01	96.25 ± 4.94****	−51.45
Group VI	MREt (30 mg/kg)	197.50 ± 6.2	105 ± 12.39****	−46.84
Group VII	MREt (60 mg/kg)	195.2 ± 5.66	98 ± 6.54****	−49.80

Values are expressed as mean ± SEM (*n* = 6). ****P < 0.0001, when compared with group II and III at respective day.

Negative (−)/positive (+) signs represent decrease/increase in percent glycemic change at 14th day.

**Table 4:** Effect of MREt on lipid profile, alanine transaminase (ALT), and antiatherogenic index in mice.

Parameters	Group I	Group II	Group III	Group IV	Group V	Group VI	Group VII
Cholesterol (mg/dL)	141.65 ± 13.05	260.42 ± 10.18	254.11 ± 14.94	178.40 ± 15.95^∗∗∗∗a,∗∗∗b^	146.19 ± 10.01^∗∗∗∗a,b^	129.33 ± 19.34^∗∗∗∗a,b^	138.50 ± 9.29^∗∗∗∗a,b^
TG (mg/dL)	148.86 ± 2.08	161.33 ± 3.83	173.50 ± 19.05	121.15 ± 3.38^∗a,∗∗b^	128.27 ± 6.59^∗a,∗∗b^	149.12 ± 15.46	138.75 ± 10.66^∗b^
HDL-c (mg/dL)	53.08 ± 8.47	50.69 ± 7.93	45.63 ± 10.01	31.20 ± 3.59	36.72 ± 5.39	36.07 ± 7.06	37.45 ± 4.03
LDL-c (mg/dL)	66.56 ± 9.61	175.09 ± 15.76	164.64 ± 19.70	122.98 ± 18.34^∗a^	83.83 ± 7.09^∗∗a,b^	63.46 ± 23.68^∗∗∗∗a,b^	73.30 ± 8.96^∗∗∗∗a,∗∗∗b^
VLDL-c (mg/dL)	29.77 ± 0.41	32.27 ± 0.77	34.70 ± 3.81	24.23 ± 0.68^∗a,∗∗b^	25.65 ± 1.32^∗a,∗∗b^	29.82 ± 3.09	27.75 ± 2.13^∗b^
AAI (%)	37.31 ± 6.43	24.98 ± 4.62	23.67 ± 6.68	23.56 ± 6.39	33.34 ± 3.83	53.29 ± 24.04	37.54 ± 4.59
ALT (U/L)	15.63 ± 0.38	16.93 ± 0.35	16.26 ± 0.19	9.82 ± 0.11^∗∗∗∗a,b^	7.68 ± 0.4^∗∗∗∗a,b^	6.75 ± 0.21^∗∗∗∗a,b^	7.25 ± 0.23^∗∗∗∗a,b^

Values are expressed as mean ± SEM (*n* = 6). *P < 0.05, **P < 0.01, ***P < 0.001, and ****P < 0.0001, when compared with respective group II (a) and III (b).
